# Effects of psychosocial function in pediatric-onset inflammatory bowel disease during the coronavirus disease 2019 pandemic

**DOI:** 10.3389/fped.2023.955293

**Published:** 2023-02-07

**Authors:** Huihui Zhang, Yun Yang, Xixi Zhao, Huajian Hu, Jia Liu, Xue Zhan, Xiaomei Song, Hong Guo, Zhongyue Li, Xiaoqin Zhou

**Affiliations:** ^1^ Department of Gastroenterology Children's Hospital of Chongqing Medical University, National Clinical Research Center for Child Health and Disorders, Ministry of Education Key Laboratory of Child Development and Disorders, Chongqing Key Laboratory of Pediatrics, Chongqing Key Laboratory of Child Health and Nutrition, Chongqing, China; ^2^ Department of Gastroenterology, Chengdu women's and Children's Central Hospital, Sichuan, China; ^3^ Department of Gastroenterology, Chongqing General Hospital, Chongqing, China

**Keywords:** corona virus disease 2019, inflammatory bowel disease, paediatric, guardian, quality of life, psychosocial function

## Abstract

**Background and Aims:**

Research on the effect of the coronavirus disease 2019 (COVID-19) pandemic on psychosocial function in patients with pediatric-onset inflammatory bowel disease (PIBD) is limited. This study aimed to evaluate the psychological status of patients with PIBD before and during the pandemic, and the relationship between mental health and disease activity.

**Methods:**

This study was a retrospective cohort study. Statistical analyses were performed to assess the relationship between demographic, clinical data and psychological data (questionnaires) of PIBD patients before and during the epidemic. The anxiety and depression emotional status of the guardians during the pandemic were evaluated.

**Results:**

In the PIBD follow-up cohort, 42 patients(male 61.9%) were included. Female with PIBD had lower pediatric quality of life inventory(PedsQL) scores (*P* = 0.007) and higher spence children's anxiety scale(SCAS) scores (*P* = 0.038) than male. The pandemic did not have a substantial impact on PedsQL, pittsburgh sleep quality index(PSQI), SCAS, or children's depression inventory(CDI) in patients with PIBD. The self-rating anxiety scale(SAS) score, anxiety rate, self-rating depression scale(SDS) score, and depression rate of PIBD guardians were significantly higher than those of healthy controls (SAS, *P* = 0.008; SDS, *P* = 0.001).

**Conclusions:**

Female children with PIBD were more vulnerable to decreased QOL and increased anxiety than male children. The anxiety and depression status of PIBD guardians were significantly higher than those of healthy controls during the COVID-19 pandemic. But the COVID-19 pandemic did not significantly affect quality of life(QOL), sleep, anxiety, or depressive mood of patients with PIBD in our study.

## 1. Introduction

Inflammatory bowel disease (IBD) is a chronic inflammatory disorder of the gastrointestinal tract, which includes Crohn's disease (CD), ulcerative colitis (UC), and IBD-unclassified (IBD-U),the main clinical symptoms include gastrointestinal manifestations such as abdominal pain, diarrhea and hematochezia, as well as extraintestinal manifestations such as joint pain, rash and so on (1). However, the etiology and mechanism of IBD remain unclear. Pediatric-onset IBD (PIBD) is defined as IBD patients younger than 17 years of age at diagnosis (2), wherein the incidence has increased significantly in recent years (3). The estimated incidence in Asia and the Middle East has varied from 0.5 to 11.4/100,000 person per year, while in mainland China, PIBD, pediatric CD (pCD) and pediatric UC (pUC) were 0.55/100,000, 0.29/100,000, and 0.25/100,000, respectively (4, 5). PIBD has a different phenotype from adult IBD, especially in young children, which is typically more aggressive and extensive at onset and is often associated with more severe complications, such as malnutrition, psychological comorbidities, and even surgery (6). Therefore, attention to PIBD and its comorbidities is important.

The coronavirus disease 2019 (COVID-19) pandemic has caused grim repercussions worldwide. The pandemic has claimed the lives of more than 5.33 million people worldwide as of December 2021 (7). Influenced by the pandemic, many Chinese hospitals have reduced outpatient services to control the spread of COVID-19 in the early stages of the outbreak (8). Under the double attack of disease and the pandemic, patients' physical, psychological, emotional, and social relationships may face huge challenges (9). Due to the chronic and recurrent characteristics of IBD itself, PIBD is often associated with more psychological and social complications (10), and is more vulnerable to a decrease in quality of life (QOL), anxiety, depression, sleep disorder, and social dysfunction. During the COVID-19 pandemic, factors such as poor access to medical care, fear of disease, and panic of the pandemic may have had an impact on emotions and QOL (11–13). However, research on this topic is limited.

In this study, we assessed the psychological status of patients with PIBD before and during the epidemic to understand the impact of the epidemic on the psychosocial function of patients with PIBD and their caregivers, as well as to evaluate the relationship between mental health and disease activity. We aimed to understand the correlation between the COVID-19 pandemic and the psychosocial and disease activity of PIBD.

## 2. Materials and methods

### 2.1. Participants

Patients were recruited through the Department of Gastroenterology of the Children's Hospital of Chongqing Medical University between January 2019 and April 2020.

PIBD diagnostic criteria was set according to the expert consensus and guideline on the diagnosis and management of pediatric IBD (14, 15).The Paris classification was applied to the disease diagnosis phenotype (2).Written informed consent was obtained from all patients, and the study was approved by the Ethics Committee of the Children's Hospital of Chongqing Medical University(approved by March, 2020).

Inclusion criteria: ①Pediatric-onset inflammatory bowel disease (<17 years old); ②Disease duration 3 years*,* inpatient setting, and ability to understand and complete questionnaires; ③The guardian as a statutory caregiver was close to the children, the included children and their caregivers were all negative for COVID-19; ④Informed consent was obtained from the patients and their guardians; ⑤The children and their caregivers completed questionnaires before the epidemic (January 2019 to December 2019) and during the epidemic (January 2020 to April 2020).

Exclusion criteria: ①With other chronic or underlying diseases that affected QOL,including heart disease, kidney disease, diabetes, mental disorders, asthma, epilepsy and other common chronic diseases in pediatrics of various systems; ②Inability to understand or complete the questionnaire; ③Age > 18 years at the time of enrollment; ④Children and their caregiver who can not finish questionnaires before and during the epidemic.

**Figure 1:**
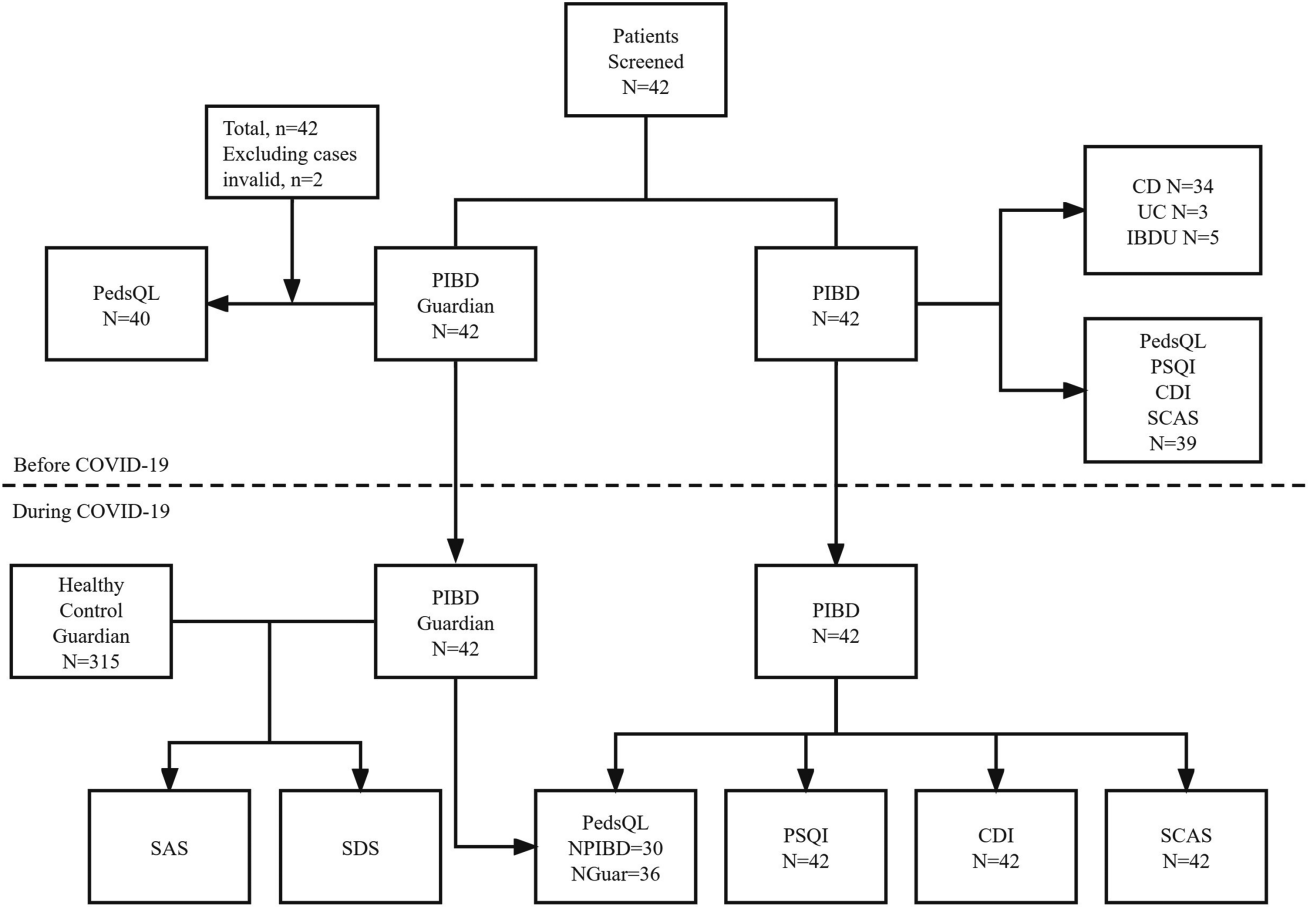
Disposition and flow of subjects in different periods.

A total of 42 patients with PIBD and 42 guardians were included. Before the epidemic, 2 invalid questionnaires were excluded, so a total of 40 patients were included. All the 40 guardians completed pediatric quality of life inventory (PedsQL) questionnaires to evaluate the QOL of PIBD patients before the epidemic, and the PIBD children independently completed questionnaires of PedsQL, Pittsburgh sleep quality index(PSQI), children's depression inventory(CDI) and spence children's anxiety scale(SCAS) (39 valid questionnaires, 1 invalid questionnaire). Meanwhile, biochemical indexes were collected and analyzed during hospitalization. After the epidemic, among the 42 PIBD children included, PSQI, CDI and SCAS were all valid questionnaires. In terms of PedsQL questionnaires, there were 30 valid questionnaires for PIBD children and 36 valid questionnaires for their guardians. In addition, 315 healthy children and guardians were included as controls and self-rating anxiety scale(SAS) and self-rating depression scale(SDS) were analyzed (Figure 1).

### 2.2. Measures

#### 2.2.1. Investigation method

Participants who agreed to participate in the survey completed the paper questionnaires before the pandemic and an online survey during the pandemic. The data included demographic information, clinical parameters, family factors, and the results of the standardized psychological tests.

#### 2.2.2. Demographic data and disease characteristics

The demographic data before the pandemic which was completed by the guardian, included sex, age, grade, relationship with the patient with PIBD, residence, family income, and guardian education level. Data regarding the disease characteristics, including IBD type, disease awareness, disease concern degree, treatment cost, past medical history, family medical history from paper questionnaires, and clinical features, were also obtained from medical record. Epidemic-related investigations were added to the duration-epidemic scales.

#### 2.2.3. Questionnaires

Guardians were required to complete the questionnaires before and during the pandemic in order to reflect the QOL of the patients. This included completion of the SAS and SDS to assess their own anxiety and depression during the pandemic. Meanwhile, patients with PIBD were also advised to complete four self-administered questionnaires before and during the pandemic, including PedsQL to evaluate QOL, PSQI, SCAS, and CDI to assess sleep, anxiety, and depression, respectively.

##### 2.2.3.1. Pediatric quality of life inventory (pedsQL)

This scale was edited by James in 1999 (16). The PedsQL version 4.0 (PedsQL 4.0), a generic core scale, is the most widely used scale to assess QOL in children with or without disease over the last month. It is a 23-item self-report tool rated on a 5-point Likert-type scale ranging from “never” = 100 to “always” = 0. It consists of four subscales: emotional, social, physiological, and role (school) functioning. Higher scores indicate better QOL. In this study, the patients' reliability coefficients were 0.909 and 0.952 before and during the pandemic, respectively, and the guardians' reliability coefficients were 0.909 and 0.952 before and during the pandemic, respectively.

##### 2.2.3.2. Pittsburgh sleep quality Index (PSQI)

The PSQI, edited by Buysse (17) in 1989, is a commonly used self-report measure of sleep quality over the last month. It is comprised of 24 items with seven components: (a) sleep quality, (b) sleep latency, (c) sleep duration, (d) sleep efficiency, (e) sleep disturbance, (f) sleeping mediations, and (g) daytime dysfunction. Each score ranges from 0 to 3, with a total score ranging from 0 to 21. Higher scores indicate poorer sleep quality. Usually, a score > 7 indicates a sleep disorder, while a score > 10 indicates very poor sleep quality. In this study, the reliability coefficients were 0.609 and 0.907 before and during the pandemic, respectively.

##### 2.2.3.3. The spence Children's anxiety scale (SCAS)

The SCAS is a 44-item self-report questionnaire that assesses multiple symptoms of childhood anxiety disorders, as edited by Spence (18). The SCAS can be used to assess overall anxiety levels, which includes anxiety symptoms related to separation anxiety disorder (6 items), obsessive-compulsive disorder (6 items), social phobia (6 items), panic attack and agoraphobia (9 items), generalized anxiety/overanxious disorder (6 items), physical injury fears (5 items), and positive/filler items (6 items), which serve to reduce negative response bias. All items are rated on a 4-point Likert-type scale in terms of its frequency from “never” = 0 to “always” = 3. Higher scores indicate higher levels of anxiety. In this study, the reliability coefficients were 0.887 and 0.967 before and during the pandemic, respectively.

##### 2.2.3.4. Children's depression inventory (CDI)

CDI is a commonly used self-report measure of depression symptoms that was edited by Kovacs in 1981 (19). The scale is comprised of 27 items on sadness, self-blame, loss of appetite, insomnia, interpersonal relationships, and school adjustment. The items were rated on a 3-point Likert-type scale (“not true” = 0, “somewhat true” = 1, “very true” = 2), reflecting the degree of particular depression symptoms over the past two weeks. The total score ranges from 0 to 54. Higher scores indicate higher levels of depression. In this study, the reliability coefficients were 0.706 and 0.979 before and during the pandemic, respectively.

##### 2.2.3.5. Self-rating anxiety scale (SAS)

The SAS is a 20-item scale used to evaluate the existence and degree of anxiety in adults within the past week (20). All items were rated on a 4-point Likert-type scale from “none or little” = 1 to “most or always” = 4, wherein 5 items were scored in reverse. The total score ranges from 20 to 100. Higher scores indicate increased anxiety. In this study, a standard score of more than 50 in SAS was considered as anxiety status, and the reliability coefficients were 0.899 and 0.813 before and during the pandemic, respectively.

##### 2.2.3.6. Self-rating depression scale (SDS)

The SDS is similar to SAS, which evaluates the presence and degree of depression in adults within the past week (21). All items were rated from 1 to 4, but 10 items needed to be scored in reverse. The scores ranged from 20 to 100, with higher scores indicating an increased level of depression. The reliability coefficients were 0.847 and 0.869 before and during the pandemic, respectively.

### 2.3. Statistical analysis

All the data were exported to Excel and SPSS statistics version 26.0. Statistical analyses were performed using the SPSS, and Graph Pad Prism 8 was used for mapping. Summary statistics for continuous variables are presented as mean ± standard deviation (SD) and compared using t-test or analysis of variance (ANOVA). Categorical data are presented as absolute (n) and relative (%) frequencies, and were compared using Fisher's exact test. Correlations were assessed by Pearson's correlation coefficient (r), or Spearman's rho. The reliability of scales used the Cronbach's alpha coefficient, wherein 0.70 represented poor, between 0.70–0.80 represented acceptable, between 0.80–0.90 represented good, and over 0.90 represented an excellent diagnostic accuracy. All scales had high internal reliability (*α* ≥ 0.8). Single-factor and multiple regression analyses were performed to explore the independent factors influencing the QOL of COVID-19 patients with PIBD. Statistical significance was set at *P* < 0.05.

## 3. Results

### 3.1. Demographic and clinical characteristics

In the PIBD follow-up cohort, a total of 40 patients were included in the demographic analysis before the COVID-19 pandemic. The mean age was 12.05 ± 2.14 years old, and the male/female ratio of patients with PIBD was 3:2, and secondary school students accounted for 60.0%. Parents were the only caregivers, and their economic income was distributed between 300 and 800 dollar per month. Sixty five percent of the parents had an education level below high school. In terms of knowledge of IBD, as much as 90% of the caregivers expressed awareness of IBD, and all showed concern about the disease.

In terms of disease types, there were 34 patients with CD, 3 with UC and 3 with IBD-U. In 34 children with Crohn's disease, A1b (10− < 17 years old) accounted for 85.3%, A1a (<10 years old) accounted for 14.7%, and the disease extent was L1 14.7%, L2 14.7%, L3 50%, L4b 5.9%, L3 + L4 11.8%, L2 + L4 2.9%. The disease behaviors of B1 accounted for 67.6%, B2 26.5%, B3 2.9% and B2B3 2.9%. The growth retardation rate was 85%. The positivity rate of perianal disease was 55.9%. All 3 patients with UC were severe. PCDAI analysis showed that 23.53% of the patients had severe disease. 40% of patients had a course of disease longer than 1 year. Abdominal pain was the most common clinical manifestation accounting for 75%, while diarrhea was the second most common at 57.5%. Through the analysis of biochemical indexes of enrolled patients, it was found that the indexes reflecting nutritional status, including Hb, Hct and PA, were lower than the normal reference value, while the indexes representing inflammatory status, including ESR, CRP and PLT, were higher than normal reference values (Table 1).

**Table 1:** Demographic and clinical characteristics of PIBD patients.

Variable	*n*/*N* (%)	Variable	*n*/*N* (%)	
Gender		PCDAI (mean ± SD) 28.15 ± 19.91		
Male	24/40 (60.00)		Remission	8/34 (23.53)
Female	16/40 (40.00)		Mild	7/34 (20.59)
		Moderate	11/34 (32.35)
Age (year) (mean ± SD)	12.05 ± 2.14		Severe	8/34 (23.53)
Guardian		Duration of disease (m) mean (IQR) 272 (90–804)		
Parents	40/40 (100.00)		<3 m	5/40 (12.50)
Grandparents	0/40 (0.00)		3 m–6 m	13/40 (32.50)
Residence			6 m–12 m	6/40 (15.00)
Rural	20/40 (50.00)		>12 m	16/40 (40.00)
Urban	20/40 (50.00)	Clinical manifestation		
Family incomes (dollar/month)			Diarrhea	23/40 (57.50)
>1,600/m	2/40 (5.00)		Abdominal pain	30/40 (75.00)
800–1,600/m	9/40 (22.50)		Fever	15/40 (37.50)
300–800/m	21/40 (52.50)		Bloody stool	11/40 (27.50)
<300/m	8/40 (20.00)		Tarry stool	2/40 (5.00)
Treatment cost (dollar)			5/40 (12.50)
>16,000	5/33 (15.15)		Unformed stool	9/40 (22.50)
8,000–16,000	3/33 (9.09)		Vomiting	3/40 (7.50)
1,600–8,000	17/33 (51.51)		Oral ulcer	8/40 (20.00)
<1,600	8/33 (24.24)		Fatigue	6/40 (15.00)
Guardian education			Weight loss	6/40 (15.00)
Bachelor	4/40 (10.00)		Others	3/40 (7.50)
Junior college	5/40 (12.50)	Nutritive index		
Senior high school	5/40 (12.50)	Hb (Mean ± SD/N)	102.45 ± 18.42/40
Under high school	26/40 (65.00)		Normal	6/40 (15.00)
			Mild decline	23/40 (57.50)
Know about IBD			Moderate decline	11/40 (27.50)
Yes	36/40 (90.00)	Hct (Mean ± SD/N)	32.57 ± 4.61/40
No	4/40 (10.00)		Normal	6/40 (15.00)
			Decline	34/40 (85.00)
Worry about the children's condition		ALB Mean (IQR)/N	38 (31.5–43.75)/40
			Normal	26/40 (65.00)
Yes	40/40 (100.00)		Decline	14/40 (35.00)
No	0/40 (0.00)	PA (Mean ± SD/N)	145.09 ± 57.86/32
			Normal	11/32 (34.38)
Past medical history			Mild decline	11/32 (34.38)
Yes	35/40 (87.50)		Moderate decline	8/32 (25.00)
No	5/40 (12.50)		Unclassified decline	2/32 (6.25)
Family medical history		Inflammatory indicator		
Yes	9/40 (22.50)	ESR (Mean ± SD/N)	51.80 ± 35.4/40
			Normal	15/40 (37.50)
No	31/40 (77.50)		Elevate	25/40 (62.50)
Disease type		CRP Mean(IQR)/N	28 (15–43)/40
			Normal	11/40 (27.50)
CD	34/40 (85.00)		Elevate	29/40 (72.50)
UC	3/40 (7.50)		Normal	8/40 (20.00)
IBD-U	3/40 (7.50)		Elevate	32/40 (80.00)

PCDAI, Pediatric Crohn's Disease Activity Index; Crohn's Disease, CD; UC, Ulcerative colitis; IBDU, IBD-unclassified; m, month; Hb, Hemoglobin; Hct, Hematocrit; PA, Prealbumin; ESR, Erythrocyte sedimentation rate; CRP, C-reactive protein; PLT, Blood platelet; IQR, range interquartile;SD, standard error.

### 3.2. Psychosocial function and clinical data

**Figure 2:**
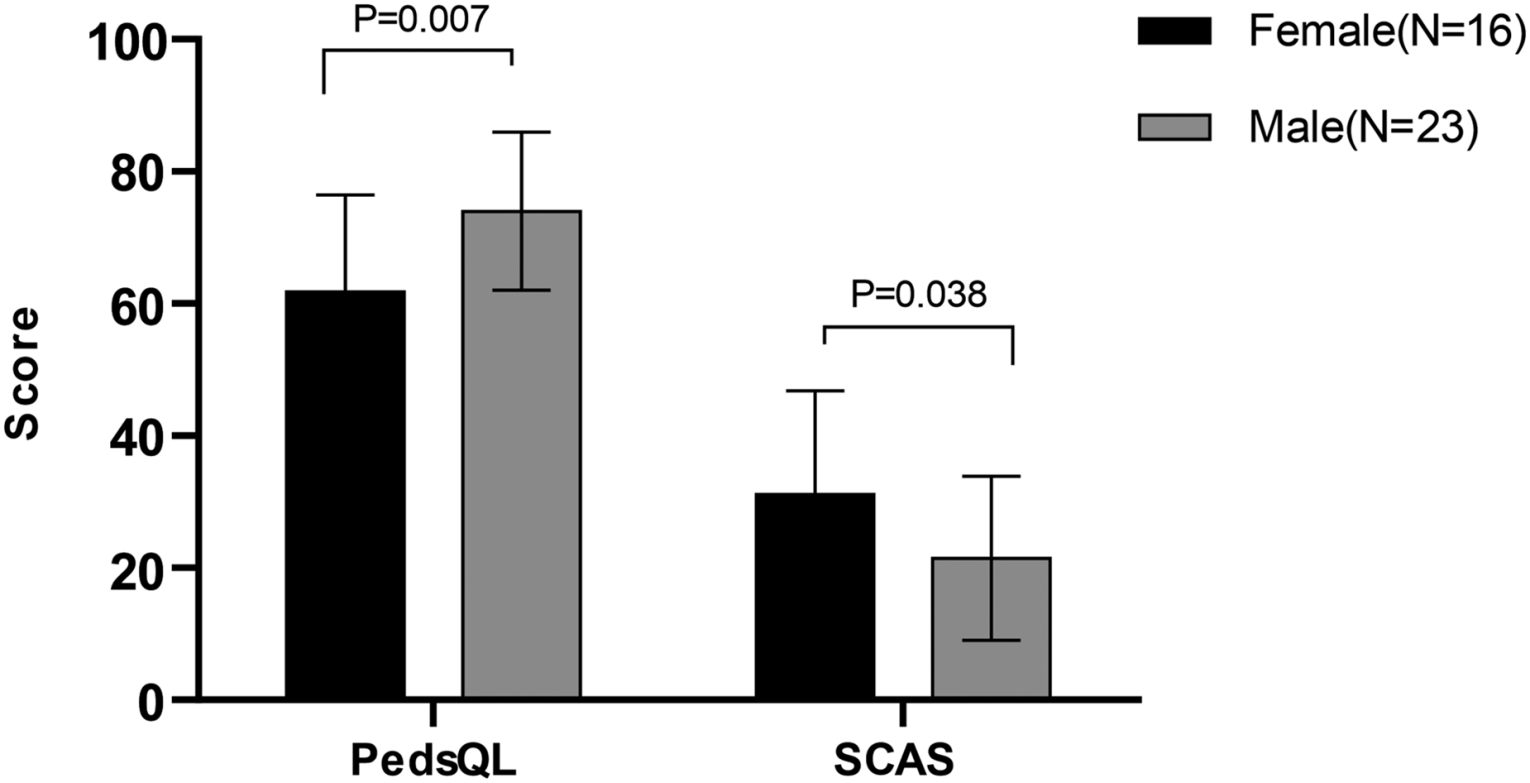
The PedsQL 4.0 and SCAS scores in different gender.

Female patients (*n* = 16) with PIBD had lower PedsQL scores (61.82 ± 14.60 vs. 73.96 ± 11.96, *P* = 0.007) and higher SCAS scores (31.13 ± 15.63 vs. 21.43 ± 12.43, *P* = 0.038) than male patients (*n* = 23), indicating that female patients with PIBD were more prone to decreased quality of life and increased anxiety than male children (Figure 2). Meanwhile, correlation analyses of clinical characteristics, and PSQI, CDI and SCAS scales were conducted. Supplementary Table S1 presents clinically meaningful results: CRP was positively correlated with daytime dysfunction in the PSQI (r = 0.606, *P* = 0.01) and the course of disease was positively correlated with sleep quality (r = 0.413, *P* = 0.045). In addition, the course of the disease was related to interpersonal problems on the CDI scale (r = 0.636, *P* = 0.001) and panic/agoraphobia on the SCAS scale (r = 0.807, *P* < 0.001). Vomiting symptoms were positively correlated with generalized anxiety (r = 0.414, *P* = 0.049). These demonstrated that the inflammatory index CRP, course of disease, and vomiting were associated with psychosocial function.

### 3.3. Psychosocial function and COVID-19 pandemic

The PedsQL scale was employed to assess the QOL of patients with PIBD before and during the COVID-19 pandemic. The QOL was better reflected due to having two perspectives, which are the patients' self-evaluation and the parents' evaluation of their children. Parents' evaluations of their children's QOL were consistent with the patients' self-evaluations. In terms of the patients' self-assessment results, each item including physiological (before 72.2 ± 18.22 vs. during78.75 ± 16.19, *P* = 0.125), emotional (66.03 ± 17.7 vs. 71.33 ± 19.56, *P* = 0.242), social (78.72 ± 16.33 vs. 83.5 ± 16.2, *P* = 0.231), role function (57.05 ± 16.69 vs. 68.83 ± 15.41, *P* = 0.004), and total score (68.98 ± 14.27 vs. 76.02 ± 14.22, *P* = 0.046) were higher during the pandemic period than before. This indicated that the pandemic did not have a substantial impact on the QOL of patients with PIBD, and even from viewing the scores, patients with PIBD scored better during the pandemic, especially in the role functional area (Table 2 and Supplementary Figure S1).

**Table 2:** The difference analysis of pedsQL 4.0 before and during COVID-19.

	N	Physiological	Emotional	Social	Role	PedsQL
**PIBD**						
Before	39	72.2 ± 18.22	66.03 ± 17.7	78.72 ± 16.33	57.05 ± 16.69	68.98 ± 14.27
During	30	78.75 ± 16.19	71.33 ± 19.56	83.5 ± 16.2	68.83 ± 15.41	76.02 ± 14.22
t		−1.554	−1.179	−1.21	−3.005	−2.034
*P*		0.125	0.242	0.231	0.004	0.046
**Guardian**						
Before	40	71.33 ± 17.72	61.38 ± 16.72	76.50 ± 17.48	58.88 ± 20.02	67.58 ± 15.12
During	36	79.52 ± 14.72	68.75 ± 17.29	85.28 ± 12.42	69.03 ± 13.14	76.15 ± 11.45
t		−2.177	−1.889	−2.498	−2.583	−2.761
*P*		0.033	0.063	0.015	0.012	0.007

PedsQL, Pediatric quality of life inventory; COVID-19, Corona virus disease 2019; PIBD, pediatric inflammatory bowel disease.

*P* < 0.05 was considered statistically significant.

The PSQI, CDI, and SCAS scales were used to evaluate sleep, depression, and anxiety, respectively, before and during the pandemic. In the aspect of sleep, PSQI scale analysis showed that patients with PIBD generally had a good sleep state, and there was no difference between before and during the epidemic period (3.87 ± 1.95 vs. 3.53 ± 1.94, *P* = 0.483). Additionally, their sleep quality (0.60 ± 0.62 vs. 1.03 ± 0.64, *P* = 0.007) and sleep disturbance (0.77 ± 0.50 vs. 0.97 ± 0.28, *P* = 0.05) scores during the pandemic were lower than those before the pandemic. Similarly, there was no increase in depression (12.50 ± 6.16 vs. 9.43 ± 6.71, *P* = 0.054) or anxiety (25.37 ± 14.66 vs. 25.31 ± 15.25, *P* = 0.987) among patients with PIBD during the pandemic. The region of negative mood (3.61 ± 1.98 vs. 2.2 ± 2.02, *P* = 0.005) and ineffectiveness (2.84 ± 1.73 vs. 1.77 ± 1.59, *P* = 0.01) displayed lower scores during the pandemic than those before the pandemic. Overall, the COVID-19 lockdown had nearly no negative impact on sleep, depression, and anxiety in the study participants (Supplementary Table S2).

Correlation analysis was conducted on the PedsQL, PSQI, CDI, and SCAS. The PedsQL score was negatively correlated with PSQI and CDI (r = −0.415 to −0.519, *P* < 0.01), and CDI was positively correlated with SCAS (r = 0.586, *P* < 0.01) before the pandemic. Analysis of the four scales during the pandemic showed that the PedsQL score was also negatively correlated to PSQI, CDI, and SCAS (r = −0.483 to −0.653, *P* < 0.01). PSQI was positively correlated with CDI and SCAS (r = 0.468 to 0.484, *P* < 0.01), and CDI was positively correlated with SCAS (r = 0.495, *P* < 0.01), suggesting that QOL, sleep, anxiety, and depression influence each other in patients with PIBD (Supplementary Table S3).

### 3.4. Psychosocial function, COVID-19 pandemic and disease activity

To further analyze the relationship between psychosocial function, the COVID-19 pandemic, and disease activity in patients with PIBD, we analyzed the delays in their treatment and medical care due to the epidemic. A total of 15 patients were treated with biological therapy, among which 5 patients had delayed treatment due to the epidemic, with the longest delay time of 3 weeks. All patients had no recurrence of disease and needed to return to hospital for re-examination during the epidemic period, so they could not seek medical treatment. Meanwhile, the patients with PCDAI scores before and during the epidemic as well as the data of psychological assessment (including QOL, PSQI, SCAS, and CDI) were included for further analysis.Thirteen patients were eligible, biochemical indexes were analyzed before and after the epidemic, and nutritional indexes including Hb(102.61 ± 18.58 vs. 120.23 ± 19.10, *P* = 0.001), Hct(32.99 ± 4.07 vs. 36.32 ± 4.67, *P* = 0.012), ALB(34.92 ± 6.50 vs. 43.52 ± 4.86, *P* = 0.01) were significantly increased after the epidemic, while inflammatory indexes including PLT(429 ± 138.25 vs. 255.23 ± 124.49, *P* = 0.001), CRP(30.85 ± 18.82 vs. 12 ± 10.14, *P* = 0.002) and ESR(65.46 ± 41.72 vs. 29.2 ± 25.27, *P* = 0.059) were significantly decreased, demonstrating their disease status improved.

According to the course of disease, there were 5 patients with the course of disease less than 12 months (Group A) and 8 patients with the course of disease more than 12 months (Group B). PCDAI, PedsQL, PSQI, SCAS and CDI were evaluated before and during the epidemic. The disease activity of both group A (pre-PCDAI 43.90 ± 13.89 vs. dur-PCDAI 16.00 ± 17.55, *P* = 0.063) and group B (40.94 ± 14.57 vs. 14.31 ± 16.01, *P* = 0.001) were significantly lower after the epidemic than before, the difference between the two groups was not statistically significant (*P*-pre = 0.724, *P*-dur = 0.862). Also, further analysis of changes in PCDAI between the two groups before and during the epidemic showed no statistical significance (*Δ*PCDAI group A −27.9 ± 24.38 vs. group B −26.625 ± 12.69, *P* = 0.903), suggesting that although the course of disease was different, the disease control status of the two groups was basically the same during the epidemic period. The PedsQL of the two groups were analyzed and found that the scores during-COVID-19 were higher than those before the epidemic, and there was no statistically significant difference between the two groups (*P*-Pre = 0.972, *P*-dur = 0.884), suggesting that the influence of COVID-19 epidemic and course of disease on QOL may not be as great as that of disease activity degree. The score changes of PSQI (group A *P* = 0.21, group B *P* = 1), SCAS(group A *P* = 0.58, group B *P* = 0.44) and CDI (group A *P* = 0.60, group B *P* = 0.55) in the two groups before and during the epidemic showed no statistical difference. However, we found that group B scored lower in all the three questionnaires than group A before the epidemic period, but higher than group A during the epidemic period, and the difference values of PSQI(*Δ*PSQI), SCAS(*Δ*SCAS) and CDI(*Δ*CDI) also showed that group B was higher than that of group A(according to PSQI, SCAS and CDI scoring criteria, higher scores were associated with worse sleep and more anxiety and depression), suggesting that the sleep, anxiety and depression of PIBD patients with long course of disease may be more susceptible to COVID-19 epidemic (Table 3).

**Table 3:** Relationship between changes in disease activity and pedsQL, PSQI, SCAS and CDI before and during COVID-19 epidemic.

Group	Group A (course of disease < 12 m) (*N* = 5)		Group B (course of disease > 12 m) (*N* = 8)		*P*
	Pre-COVID-19	During-COVID-19	Pre-COVID-19	During-COVID-19	
PCDAI	43.90 ± 13.89	16.00 ± 17.55	40.94 ± 14.57	14.31 ± 16.01	*P*-pre = 0.724
(mean ± SD)					*P*-dur = 0.862
*P*	*P* = 0.063	*P* = 0.001	
ΔPCDAI	−27.9 ± 24.38	−26.625 ± 12.69	0.903
PedsQL	69.84 ± 19.17	82.88 ± 19.41	70.29 ± 10.67	84.78 ± 9.66	*P*-pre = 0.972
(mean ± SD)					*P*-dur = 0.884
*P*	*P* = 0.023	*P* = 0.311	
ΔPedsQL	13.05 ± 6.08	14.49 ± 18.65	0.887
PSQI	5.00 ± 1.41	3.25 ± 1.50	4.33 ± 1.15	4.33 ± 2.52	*P*-pre = 0.537
(mean ± SD)					*P*-dur = 0.504
*P*	*P* = 0.213	*P* = 1	
ΔPSQI	−1.75 ± 2.22	0 ± 1.73	0.312
SCAS	26.50 ± 19.94	20.00 ± 11.22	25 ± 16.43	33.75 ± 16.19	*P*-pre = 0.911
(mean ± SD)					*P*-dur = 0.212
*P*	*P* = 0.58	*P* = 0.437	
ΔSCAS	−6.5 ± 21.01	8.75 ± 19.59	0.329
CDI	12.00 ± 6.06	8.50 ± 9.75	8.67 ± 5.51	12.33 ± 5.03	*P*-pre = 0.489
(mean ± SD)					*P*-dur = 0.567
*P*	*P* = 0.604	*P* = 0.552	
ΔCDI	−3.5 ± 12.12	3.67 ± 8.96	0.431

PedsQL, pediatric quality of life inventory; PSQI, *p*ittsburgh sleep quality Index; CDI, Children's depression inventory; SCAS, the Spence Children's anxiety scale; COVID-19, corona virus disease 2019; *P* < 0.05 was considered statistically significant.

### 3.5. Family functioning and COVID-19 pandemic

In order to understand the influence of family function in patients with PIBD during the pandemic, PIBD (*N* = 42) and healthy controls (*N* = 315) were included, and their general information and parents' attention during the pandemic were analyzed. The male/female ratios of the two groups were 13:8 and 5:4, respectively. Of the children in the healthy group, 51.75% were 6–11 years old, while 76.19% of the children in the PIBD group were older than 11 years old. More than 95% of the caregivers were parents, wherein their living environment was mainly rural, and the family income of patients with PIBD were low, accounting for 57.14% with annual income < 5,000 dollars, while the family income of healthy controls ranged from 25,000 dollars to 130,000 dollars. In terms of the educational background of the parents, parents of PIBD children mainly have junior high or below degree, and their occupation type is mainly farmers, while parents of healthy control mainly have bachelor degree, and their occupation type is mainly enterprise employees. In terms of attention time to the epidemic, the parents of the two groups mainly paid attention to the epidemic three minutes to one hour per day. WeChat was chosen as the most commonly used information transmission tool in the two groups (69.05% and 71.43%, respectively). In terms of the parents’ main stress and worry, the guardians of patients with PIBD mainly expressed their worries about the disease and medical treatment, while the parents of healthy controls mainly expressed their worries about not being able to go to school and live a normal life (Table 4).

**Table 4:** The general information of PIBD and healthy controls after COVID-19 (N_PIBD_ = 42, n_healthy_ = 315).

Variable	PIBD Group	Healthy controls	Variable	PIBD Group	Healthy controls
	*N* (%)	*N* (%)		*N*(%)	*N*(%)
Gender			Freelancer	11 (26.19)	42 (13.33)
Male	26 (61.90)	175 (55.56)	Retiree	0	3 (0.95)
Female	16 (38.10)	140 (44.44)	Farmers	14 (33.33)	2 (0.63)
Age (year)			Soldier	0	2 (0.63)
<1 year	0	5 (1.59)	Others	1 (2.38)	11 (3.49)
1–3 year	3 (7.14)	26 (8.25)	Length of viewing news of COVID-19 (guardian)		
3–6 year	0	74(23.49)	Unconcern	1(2.38)	1(0.32)
6–11 year	7 (16.67)	163 (51.75)	<10 min	15 (35.71)	56 (17.88)
>11 year	32 (76.19)	47 (14.92)	3 min-1 h	21 (50.00)	115 (36.51)
Guardian			1–3 h	3 (7.14)	89 (28.25)
Parents	40 (95.24)	306 (97.14)	>3 h	2 (4.76)	45 (14.29)
Grandparents	1 (2.38)	4 (1.27)	Unclear	0	9 (2.86)
Others	1 (2.38)	5 (1.59)	Tools for viewing epidemic information (guardian)		
Residence			Mricroblog	4(9.52)	66 (20.95)
Urban	8 (19.05)	2 (0.63)	WeChat	29 (69.05)	225 (71.43)
Rural	21 (50.00)	288 (91.43)	QQ	6 (14.29)	8 (2.54)
Suburb	13 (31.95)	25 (7.94)	Short video	14 (33.33)	58 (18.41)
Family incomes (dollar/year)			Relatives and friends	8 (19.05)	31 (9.84)
<5,000	24 (57.14)	8 (2.54)	Television news	25 (59.52)	148 (46.98)
5,000–13,000	15 (35.71)	55 (17.46)	Colleague	4 (9.52)	11 (3.49)
13,000–25,000	3 (7.14)	120 (38.10)	Network	26 (61.90)	196 (62.22)
25,00–130,000	0	125 (39.68)	Others	3 (7.14)	7 (2.22)
>130,000	0	7 (2.22)	Main pressure (guardian)		
Guardian education			COVID-19	1 (2.38)	33 (10.48)
Postgraduate or above	0	37 (11.75)	IBD	19 (45.24)	48 (15.24)
Undergraduate	8 (19.05)	162 (51.43)	COVID-19 + other disease	19 (45.24)	175 (55.56)
Junior college (Higher vocational)	3(7.14)	68 (21.59)	Life stress	3 (7.14)	35 (11.11)
High school (technical secondary)	9(21.43)	34 (10.79)	Family marital stress	0	8 (2.54)
Junior high or below	22 (52.38)	14 (4.44)	Others	0	16 (5.08)
Occupation (guardian)			Worry about (guardian)		
Civil servants	1 (2.38)	15 (4.76)	Infection with the COVID-19	6 (14.29)	101 (32.06)
Public institution	5 (11.90)	83 (26.35)	Unable to timely treatment, disease recurrence or aggravation	22 (52.38)	28 (8.89)
Employees of enterprises	6 (14.29)	108 (34.29)	Unable to go out to study and live normally	14 (33.33)	172 (54.60)
Self-employed person	4 (9.52)	49 (15.56)	Unclear	0	14 (4.44)

COVID-19, corona virus 2019; y, year; IBD, inflammatory bowel disease.

Anxiety and depression among caregivers in the two groups were analyzed. The SAS questionnaire was used to assess anxiety levels. It was found that the SAS score of the guardians of the patients with PIBD (44.29 ± 13.41) was significantly higher than that of the guardians of healthy controls (38.39 ± 8.12), and the anxiety rate of the guardians of the patients with PIBD (31%) was significantly higher than that of the guardians of healthy controls (10.2%) (*P* = 0.008). The SDS questionnaire was used to evaluate the depression status of the guardians, wherein the SDS scores of the guardians of the patients with PIBD (47.29 ± 12.05) were also significantly higher than those of the guardians of healthy controls (40.63 ± 12.20). The depression rates of the two groups were 43% and 19%, respectively (*P* = 0.001), indicating that the anxiety and depression of the guardians of the patients with PIBD were significantly higher than those of the guardians of healthy children under the pandemic stress factors (Table 5).

**Table 5:** The SAS and SDS of guardians during COVID-19.

Group	Variable	Mean ± SD	Positive^#^	*N*	%	*P*
Healthy controls guardians	SAS	38.39 ± 8.12	No	283	89.80%	0.008^&^
			Yes	32	10.20%	
	SDS	40.63 ± 12.20	No	255	81.00%	0.001*
			Yes	60	19.00%	
PIBD guardians	SAS	44.29 ± 13.41	No	29	69.00%	0.008
			Yes	13	31.00%	
	SDS	47.29 ± 12.05	No	24	57.00%	0.001
			Yes	18	43.00%	

SAS, Self-rating Anxiety Scale;SDS, Self-rating Depression Scale; SD, standard deviation;COVID-19, Corona virus disease 2019; PIBD, pediatric inflammatory bowel disease.

^#^ According to the results of SAS and SDS Chinese norm standard, the standard score is divided into anxiety threshold of 50, depression threshold of 50, and the score greater than the threshold is yes, less than the threshold is no.

^&^ SAS, Healthy controls guardians vs. PIBD guardians, *P* = 0.008.

* SDS, Healthy controls guardians vs. PIBD guardians, *P* = 0.001.

## 4. Discussion

This study mainly focused on the psychosocial function of patients with PIBD and found that female children with PIBD were more vulnerable than male children in terms of QOL and anxiety. However, the pandemic factors did not have a substantial influence on QOL, sleep, anxiety, and depression in patients with PIBD, but the anxiety and depression of the guardians of the patients with PIBD under the stress of the pandemic was significantly higher than that of the guardian of healthy controls. The effects of disease activity and perianal problems on PIBD QOL, sleep, and mood warrant further investigation. To the best of our knowledge, this is the first study with data from the same group of patients before and during the pandemic and a comprehensive analysis of QOL, sleep, anxiety, depression and parental mood.

Approximately 20% of individuals with IBD are diagnosed during childhood (22). The disease leads to repeated hospitalizations, academic delays, and inconveniences in daily life, which creates potential psychological stress. In a systematic review of 12,540 patients with IBD, the prevalence of psychiatric disorders in patients with PIBD was 21.6% compared to 6% in the control group. Moreover, the incidence of mental disorders in IBD patients showed a steady and significant upward trend, and the prevalence was higher in women than in men (23). In our study, we found that the QOL and anxiety state of female children with PIBD were more obvious than those of male children, which is consistent with the findings of other research groups (24–26). According to some previous evidence, females are more concerned and sensitive to their own diseases and physical symptoms, which correlates with age, especially in adolescent and adult young women, suggesting that more attention should be paid to female patients with PIBD (27, 28).

The COVID-19 pandemic has seriously affected the population lives. In the early stage, China began home quarantine, business and public transportation shutdown, class suspension, and hospitals reduced outpatient clinics to contain the outbreak. This led to a conflict between the need for follow-up and the risk of COVID-19 in patients with IBD (29). Mental health issues during the outbreak have been reported in several studies (9, 30–32). A lockdown during the COVID-19 pandemic had a psychological impact on patients with IBD (33). In this study, we also evaluated the QOL of children with PIBD and their parents before and during the pandemic. The results of the children's self-evaluation and the guardian's evaluation were the same regardless of the pandemic. Interestingly, the total score during the pandemic was higher than before the pandemic. Moreover, the scores in the role function item in the children's self-assessment and guardian assessment were higher than those before the pandemic. In view of this phenomenon, we considered two factors. On one hand, pandemic factors weaken the specificity of some physical symptoms of PIBD, and isolation at home may normalize their social roles. However, the disease activity status of subjects may have improved after treatment. To verify this consideration, we separately analyzed the patients who had both the PCDAI score and the QOL assessment data before and during the pandemic. We found that the improvement rate of self-rated QOL in patients in remission was higher than that in patients who were still in the active stage. However, further grouping analysis was conducted according to the degree of change in disease activity and it was found that the degree of change in disease activity did not seem to have a significant relationship with QOL, but there was indeed a bias with a small sample size. Therefore, further analysis of the relationship between disease activity change, QOL, and even stress factors should be conducted by expanding the sample size. Surveys in different countries have shown that the QOL was not significantly affected by COVID-19, and the disease itself may be the main factor involved, suggesting that attention should be paid to the treatment of the primary disease (34, 35).

Anxiety and depressive mood disorders are common mood problems in IBD patients. Nearly 60% of the respondents had clinical anxiety, 40% reported depressive symptoms, and younger people were more likely to show psychological abnormalities (36). Sleep disturbances are a major problem in patients with IBD. Canadian survey of adult IBD patients showed that there was a four-fold increase in anxiety during the pandemic compared to pre-pandemic levels (37). An Australian national survey demonstrated that IBD patients experienced high levels of undiagnosed anxiety, depression, and stress during the pandemic (38). Nishida et al. found sleep hours to be an independent factor associated with IBD exacerbation during the COVID-19 pandemic (39). In this study, we found that the COVID-19 lockdown had no impact on anxiety, depression, and sleep in patients with PIBD. To analyze the reasons for this, we evaluated the pre-pandemic data of patients with PIBD and observed that CRP, course of disease, and vomiting symptoms were related to sleep, anxiety, and depressive mood, suggesting that disease activity status and physical symptoms may be related to this psychosocial function. Therefore, we also independently analyzed the data of patients with both PCDAI, PSQI, SCAS, and CDI before and during the epidemic, and found that thirteen patients were eligible; disease activity status was improved from the score of biochemical indexes and PCDAI. Group analysis by course of disease found that the influence of COVID-19 epidemic and course of disease on QOL may not be as great as that of disease activity degree. But what's interesting is that the sleep, anxiety and depression of PIBD patients with long course of disease may be more susceptible to COVID-19 epidemic, suggesting that patients with PIBD with a long course of disease may have a weaker ability to adjust to stressful events than patients with short course of disease, and more attention should be paid to the psychological status of patients with IBD with a long course of disease in clinical practice. This is consistent with a study by Conti et al., who investigated the anxiety, depression, QOL, and somatizing distressing symptoms of patients with IBD during the outbreak of COVID-19, and found that the QOL of patients with IBD was mainly affected by psychological and somatizing distressing symptoms, rather than by the pandemic (40).

Family functioning plays an important role in psychological well-being in PIBD. Caregivers of the patients with PIBD reported higher levels of psychological distress symptoms than healthy controls (41, 42).We evaluated the guardians of the patients with PIBD for anxiety and depression, and found that guardians of the patients with PIBD had higher anxiety and depression scores and negative emotion rates than guardians of healthy controls during the pandemic. In terms of attention to the pandemic, 45.2% of PIBD parents' stress came from IBD, and 52.4% of PIBD family members expressed concern about not being able to seek medical treatment on time, suggesting that anxiety and depression of PIBD family members during the COVID-19 pandemic mainly came from concerns about the disease of their children.

The strengths of this research is that it includes the same group of PIBD patients before and during the pandemic and a comprehensive analysis of the relationship between psychological impact and epidemic.The small sample size is the main limitation of this study. In addition, there is segregation, online teaching and even medical care delayed involved in the special stress events of the epidemic. Therefore, uniformity cannot be guaranteed, and there are problems of confusion and potential bias. At present, these patients are still under follow-up management and we hope to dynamically evaluate the relationship between their long-term psychological status and disease status again in the later stage, and we will pay more attention to taking into account these interfering factors in the future analysis.

## 5. Conclusion

This study mainly focused on the psychosocial function of patients with PIBD and found that female children with PIBD were more vulnerable than male children in terms of QOL and anxiety. During COVID-19, the PIBD guardians' anxiety and depression status was significantly higher than that of healthy controls. However, taking into account the sample size, we found that COVID-19 did not significantly affect the QOL, sleep, anxiety, and depressive mood of patients with PIBD.The effects of course of disease and disease activity on the QOL, sleep, and mood of patients with PIBD warrant further investigation**.**

## Data Availability

The original contributions presented in the study are included in the article/**Supplementary Material**, further inquiries can be directed to the corresponding author/s.
