# Supplementary material for: Effects of psychosocial function in pediatric-onset inflammatory bowel disease during the coronavirus disease 2019 pandemic
Source: Front Pediatr. 2023 Feb 7;11:955293. doi: 10.3389/fped.2023.955293 (PMC9941342; doi:10.3389/fped.2023.955293)
Supplement: Supplementary file 1 [file Datasheet1.pdf]

Supplementary Material

1 Supplementary Tables

Supplementary Table 1. The correlation analysis of clinical characteristics and PSQI, CDI, SCAS

Index	CRP	During of disease	Vomit
**PSQI**			
Daytime dysfunction	0.606**	-0.055	0.052
Sleep quality	0.163	0.413*	-0.015
**CDI**			
Interpersonal problem	-0.646**	0.636**	0.045
**SCAS**			
Panic/agoraphobia	-0.347	0.807**	-0.003
Generalised anxiety	0	0.087	0.414*

PSQI,Pittsburgh Sleep Quality Index; CDI,Children's Depression Inventory; SCAS, The Spence Children's Anxiety Scale; CRP, C-reactive protein; *, P＜0.05; **, P＜0.01.

Supplementary Table 2. The difference analysis of PSQI, CDI and SCAS before and during COVID-19

	Before(N=39)	During(N=42)	t	*P*
**PSQI**	3.87±1.95	3.53±1.94	0.705	0.483
Sleep quality	1.03±0.64	0.60±0.62	2.772	0.007
Sleep latency	0.66±0.75	0.80±0.66	-0.818	0.416
Sleep duration	0.13±0.53	0.13±0.35	-0.016	0.988
Sleep Efficientcy	0.24±0.63	0.33±0.66	-0.612	0.543
Sleep disturbances	0.97±0.28	0.77±0.50	2.012	0.05
Sleep Medication	0.05±0.23	0.20±0.55	-1.376	0.177
Daytime Dysfunction	0.79±0.58	0.70±0.65	0.6	0.551
**CDI**	12.50±6.16	9.43±6.71	1.959	0.054
Negative Mood	3.61±1.98	2.2±2.02	2.878	0.005
Interpersonal Problem	0.71±0.87	0.6±1.22	0.436	0.664
Ineffectiveness	2.84±1.73	1.77±1.59	2.634	0.01
Anhedonia	3.58±2.38	3.03±2.55	0.909	0.366
Negative Self-Esteem	1.76±1.38	1.83±1.21	-0.22	0.827
**SCAS**	25.37±14.66	25.31±15.25	0.016	0.987
Panic/agoraphobia	2.95±3.37	2.83±2.98	0.152	0.88
Separation anxiety	2.82±2.66	3.48±3.31	-0.914	0.364
Social phobia	5.74±4.45	5.69±4.17	0.044	0.965
Physical injury fears	4.42±2.34	4.28±3.03	0.221	0.826
Obsessive-compulsive disorder	4.45±3.18	3.76±2.39	0.976	0.333
Generalised anxiety	4.21±2.71	4.24±3.15	-0.043	0.966

PSQI,Pittsburgh Sleep Quality Index;CDI,Children's Depression Inventory;SCAS,The Spence Children's Anxiety Scale;COVID-19,Corona virus disease 2019; P＜0.05 was considered statistically significant.

Supplementary Table 3. The correlation analysis of scales before and during COVID-19 pandemic

Pearson	PedsQL	PSQI	CDI	SCAS
**Before**				
PedsQL	1			
PSQI	-.415**	1		
CDI	-.519**	0.321	1	
SCAS	-0.307	0.323	.586**	1
**During**				
PedsQL	1			
PSQI	-.524**	1		
CDI	-.653**	.468**	1	
SCAS	-.483**	.484**	.495**	1

PedsQL, Pediatric quality of life inventory; PSQI,Pittsburgh Sleep Quality Index; CDI,Children's Depression Inventory; SCAS,The Spence Children's Anxiety Scale.**, P<0.01, P＜0.05 was considered statistically significant.

2 Supplementary Figures

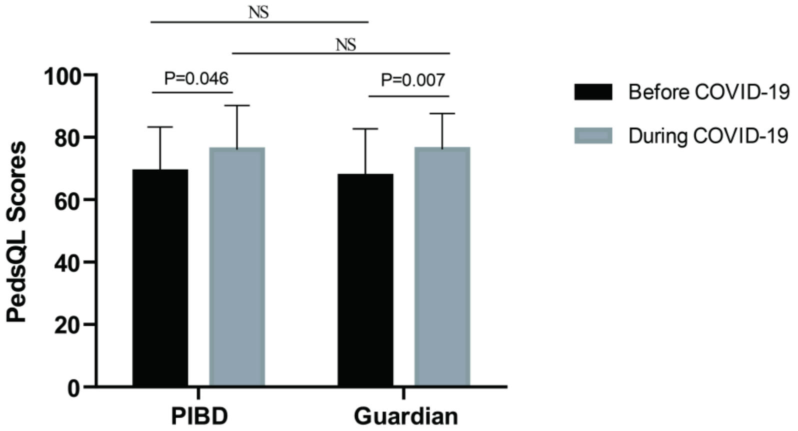

Supplementary Figure 1. The difference analysis of PedsQL before and during COVID-19 pandemic

$PIBD_{bef}$=39, $PIBD_{dur}$=42, $Guardian_{bef}$=40, $Guardian_{dur}$=36.

COVID-19, Coronavirus disease 2019; bef, before; dur, during.
